# Engineering an efficient secretion of leech carboxypeptidase inhibitor in *Escherichia coli*

**DOI:** 10.1186/1475-2859-8-57

**Published:** 2009-10-29

**Authors:** Juan-Miguel Puertas, Jean-Michel Betton

**Affiliations:** 1 Unité de Biochimie Structurale, Institut Pasteur, URA-CNRS 2185, 75724 Paris Cedex 15, France; 2 Departament d'Enginyeria Química, Universitat Autònoma de Barcelona, Bellaterra, 08193, Spain

## Abstract

**Background:**

Despite advances in expression technologies, the efficient production of heterologous secreted proteins in *Escherichia coli *remains a challenge. One frequent limitation relies on their inability to be exported to the *E. coli *periplasm. However, recent studies have suggested that translational kinetics and signal sequences act in concert to modulate the export process.

**Results:**

In order to produce leech carboxypeptidase inhibitor (LCI) in the bacterial periplasm, we compared expression of the natural and optimized gene sequences, and evaluated export efficiency of LCI fused to different signal sequences. The best combination of these factors acting on translation and export was obtained when the signal sequence of DsbA was fused to an *E. coli *codon-optimized mature LCI sequence. When tested in high cell density cultures, the protein was primarily found in the growth medium. Under these conditions, the engineered expression system yields over 470 mg.l^-1 ^of purified active LCI.

**Conclusion:**

These results support the hypothesis that heterologous secreted proteins require proper coupling between translation and translocation for optimal high-level production in *E. coli*.

## Background

*Escherichia coli *is by far the simplest, but one of the most widely used host cell for the production of recombinant proteins [1]. Nevertheless, the efficient translocation across the inner membrane and proper periplasmic folding of eukaryotic proteins stabilized by multiple disulfide bonds remains challenging for this organism [2]. Unfortunately, many proteins of which there is a great biotechnological or biomedical interest are secreted proteins containing essential disulfide bonds for their native structure. Either premature cytoplasmic protein folding or incorrect disulfide bond formation in the bacterial periplasm are two known limitations in the overproduction of secreted proteins [3]. Recently, it has been reported that signal sequences promoting co-translational translocation improved the translocation of heterologous proteins [4]. Therefore, targeting these recombinant precursors to the cotranslational signal recognition particle (SRP) dependent pathway conceivably could result in much higher levels of periplasmic proteins than directing them posttranslationally to the SecYEG translocase [5]. Strategies to overcome folding problems due to disulfide bond formation have primarily focused on the co-production of protein disulfide isomerases [3]. For example, the overproduction of DsbC, a periplasmic thiol isomerase, resulted in large amounts of native human tissue plasminogen activator [6].

In the present study, we have investigated the production of leech carboxypeptidase inhibitor (LCI) in the periplasm of *E. coli*. This protein is composed of 66 amino acid residues forming a globular domain with five-stranded β-sheet and a short α-helix that are stabilized by four disulfide bonds [7]. Like other small disulfide-rich proteins, the active conformation of LCI is strictly dependent upon the correct formation of disulfide bonds [8]. Found in the digestive track of leeches, LCI is a strong inhibitor of human pancreatic and plasma carboxypeptidases, and thus has considerable biomedical interest [9]. Indeed, by targeting the thrombin-activatable fibrinolysis inhibitor (TAFI) involved in hemostasis, LCI could play an important role in thrombotic disorder therapy [10]. The binding and inhibition activity of LCI is primarily exerted by its C-terminal extremity that interacts with the active site of metallo-carboxypeptidases. In order to overproduce LCI in the periplasm, an *E. coli *codon-optimized sequence and different signal sequences were evaluated using a tightly controlled expression vector, suitable for high cell density cultures.

## Results and Discussion

### Construction of LCI precursors

**Figure 1:**
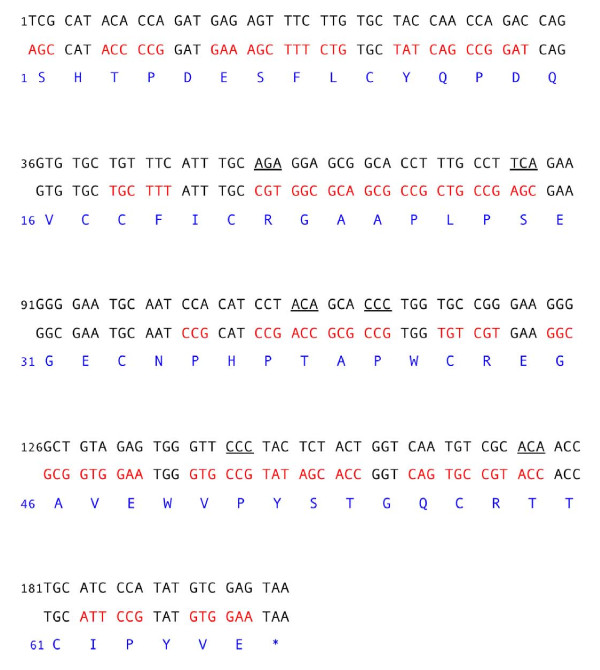
**Codon optimization of the mature LCI sequence**. The natural and *E. coli *optimized nucleotide sequences coding the mature LCI, indicated in blue, are aligned. Codons occurring at a frequency below 8‰ in *E. coli *are underlined and codon changes are indicated in red. The codon adaptation index or CAI [28] shifts from 0.209 for the natural sequence to 0.622 for the optimized sequence.

Native LCI has been previously produced in *E. coli *using the signal sequence of OmpA (OmpAss), but the low yield of secreted protein and plasmid instability precluded any development requiring large-scale production [9]. We decided to investigate several expression parameters in order to improve the production of LCI in the periplasm of *E. coli*. The gene encoding the mature LCI protein (LCI_N_), as determined from the medical leech *Hirudo medicinalis *[9], contains 7 codons that are used at a frequency below 8 per 1000 in *E. coli *[11]. We hypothesized that a biased codon usage might limit its expression level. Since other non-optimal codons for *E. coli *are also present in LCI_N_, a whole synthetic coding sequence with codon usage optimized for *E. coli *was designed. The resulting LCI_O _sequence contains 44 codon changes over the 66 codons (figure 1). Both LCI_N _and LCI_O _were subcloned under the control of the tightly regulated *araB *promoter from the pLCB vector encoding chloramphenicol resistance. This expression vector, derived from the pBAD33 plasmid [12], was constructed for achieving high cell density cultures. In a first attempt, we retained the original OmpAss to evaluate the effect of codon optimization. Next, we investigated whether the limiting expression levels were only governed by its mature sequence, and tested the production of LCI_N _and LCI_O _fused either to the signal sequence of DsbA (DsbAss) or MalE (MalEss), two well-studied periplasmic proteins of *E. coli*. We chose these signal sequences because there are known to direct co- and post-translational export of heterologous precursors through the SRP- and Sec-dependent pathways, respectively [5]. Besides translocation modes, it was recently shown that a biased codon usage in signal sequences may also play a role in the coupling of translation to protein export, by slowing down the translation rate [13]. Therefore, we decided to use the natural signal sequences of *E. coli *for targeting the various LCI precursors (preLCI) to the SecYEG translocase.

### MalEss and DsbAss promote high-level of LCI in the periplasm

**Figure 2:**
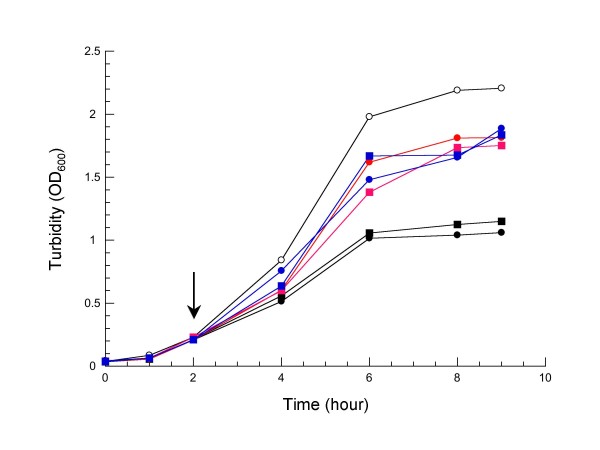
**Growth curves of *E. coli *expressing LCI in shake-flask cultures**. LMG194 cells carrying pLCB (open circle), pOmpAssLCI_N _(black circle), pOmpAssLCI_O _(black square), pDsbAssLCI_N_(red circle), pDsbALCI_O _(red square), pMalEssLCI_N _(blue circle), and pMalEssLCI_O _(blue square), were grown at 37°C in LB medium supplemented with 30 μg.ml^-1 ^chloramphenicol. Induction by arabinose (0.2% final) was performed at time indicated by an arrow.

**Figure 3:**
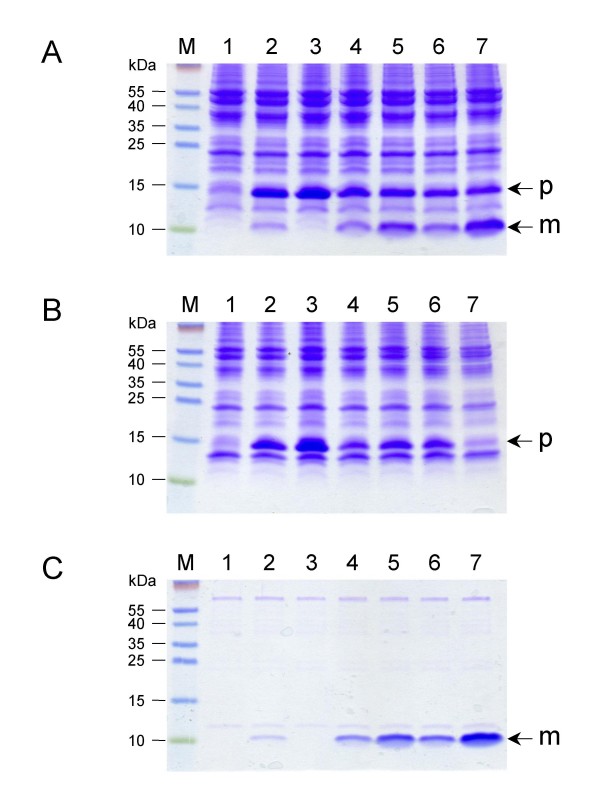
**Steady-state level and cellular location of LCI**. Whole cell lysates (A), cytoplasmic fractions (B), and periplasmic fractions (C) were separated by SDS-PAGE and stained by Coomassie blue. Lanes 1, pLCB; lanes 2, pOmpAssLCI_N_; lanes 3, pOmpAssLCI_O_; lanes 4, pMalEssLCI_N_; lanes 5, pMalEssLCI_O_; lanes 6, pDsbAssLCI_N_; lanes 7, pDsbALCI_O_. The position of precursor (p) and mature (m) LCI is indicated by an arrow.

All expression vectors were transformed into the LMG194 strain in which arabinose is not catabolized because of a deletion encompassing most of the *araBAD *operon [12]. The corresponding cells were cultured at 37°C in LB media supplemented with chloramphenicol. After 2 h of induction, LCI expression appeared to slow down cell growth progressively (figure 2). When growth stops, a higher density was achieved for cells expressing LCI fused to MalEss or DsbAss than for those expressing LCI fused to OmpAss, independent of codon optimization. This observation suggested that the physiological state of the corresponding cells could be affected. We took advantage of the carboxypeptidase inhibitory activity of LCI as a reliable indicator of active protein to detect its presence in both whole cell lysates and culture supernatants. Indeed, it was previously reported that periplasmic LCI could be released into the growth medium [9], as frequently observed for small exported proteins [14]. Table 1 shows the distribution of active LCI between cells and culture supernatants determined from the six expression vectors. Cells expressing LCI fused either to DsbAss or MalEss displayed higher levels of activity than those expressing LCI with OmpAss (Table 1). If the LCI protein is unable to fold within the reductive environment of the cytoplasm [15], these data could reflect differences in the efficiency of export of the different LCI precursors (see below). However, active LCI was found in the culture supernatants when cells expressed the *E. coli *optimized gene, suggesting also a translational effect. To assess the steady-state production and cellular location of LCI, cells were fractionated from spheroplasts. Regardless of the signal sequence used, figure 3 shows the accumulation of large amounts of preLCI for all expression vectors. In addition, expression from LCI_opt _was markedly higher than from LCI_N_, consistent with increased translation rate. Nevertheless, for cells expressing LCI fused to OmpAss, little or no protein was detectable in the periplasmic fractions while preLCIs were correctly produced. This result indicated that LCI export did not occur to any significant extent when OmpAss was used, and also confirmed previous findings [9]. It is noteworthy that growth of the corresponding cells was severely inhibited. The relative amounts of precursor (p) and mature (m) LCI were assessed, and export efficiency (m/p) evaluated for each expression vectors (Table 2). To ensure that all periplasmic contents were released during spheroplast preparation, we compared m/p ratios determined from whole cell extracts to those determined from subcellular fractions (Table 2). Because both values are similar, it can be concluded that: (i) a complete release of the periplasmic mature LCI had been attained, and (ii) all preLCIs remained in the soluble cytoplasmic fractions. Besides OmpAss, our results indicated that the two other preLCIs with optimized *E. coli *codons could give rise to a high level of periplasmic expression (figure 3C). The highest export efficiency was observed with LCI_O _fused to DsbAss. Interestingly, amounts of LCI in the culture supernatants (Table 1) correlated with their levels in the corresponding periplasmic fractions, suggesting that the presence of extracellular LCI resulted from a direct leakage of the outer membrane. Although the level of LCI when fused to MalEss was somewhat lower than when fused to DsbAss, the efficient export of preLCI may require a co-translocation mode. Since pDsbALCIo seemed to be a good expression vector, we checked its stability on selection pressure. After different cultivation times, bacteria were plated onto solid LB media with and without chloramphenicol in the presence of arabinose. After overnight growth, the number of colony forming units (CFU) determined from these plates indicated no plasmid loss (see below).

**Table 1:** Distribution of native LCI between cells and culture medium

	**pOmpALCI_N_**	**pOmpALCI_O_**	**pDsbALCI_N_**	**pDsbALCI_O_**	**pMalEssLCI_N_**	**pMalEssLCI_O_**
Whole cell lysates	53	33.5	147.5	154.5	104	125.5

Culture supernatants	nd	nd	nd	12.5	nd	4

The level of LCI (mg. l^-1^) in these fractions was assayed by a spectrophotometric assay using bovine carboxypeptidase A, and normalized to protein content of whole cell lysates determined by the Bradford procedure using bovine serum albumin as a standard (nd, not detectable).

**Table 2:** Export efficiency of preLCI

	**pOmpALCI_N_**	**pOmpALCI_O_**	**pDsbALCI_N_**	**pDsbALCI_O_**	**pMalEssLCI_N_**	**pMalEssLCI_O_**
R1	0.4	0.1	1.3	4.8	1.0	1.8

R2	0.2	0.01	1.1	5.7	0.9	1.5

The relative amount of precursor (p) and mature (m) LCI present in each gel shown in figure 3 was assessed by densitometric scanning and the ratio R (m/p) was determined from whole cell lysates (R1), and from cytoplasmic and periplasmic fractions (R2).

### High level production of LCI in a fermentor

**Figure 4:**
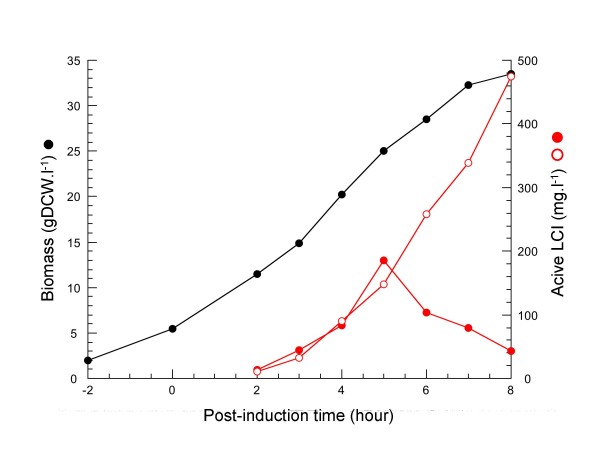
**High scale production of LCI**. The culture of LMG194/pDsbALCI_O _cells in a 2-l fermentor is shown with time course variations of biomass (gDCW.l^-1^, black circle) and distribution of active LCI (mg.l^-1^) between cells (red circle) and culture supernatants (open circle).

**Figure 5:**
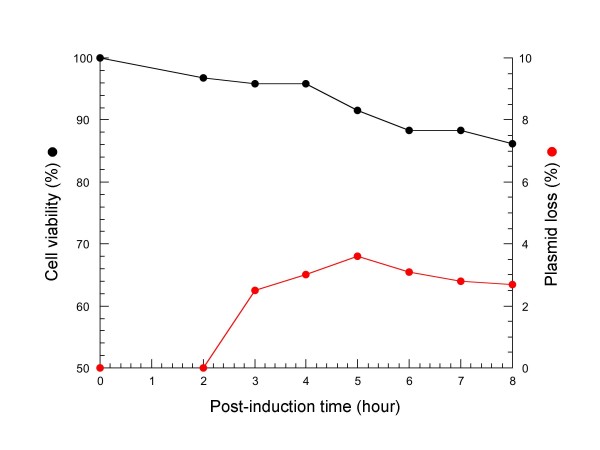
**Cell viability and plasmid stability in high cell density culture**. Duplicate samples were taken from the high cell density culture shown in figure 4. From these culture samples, cell viability (%, black circle) and plasmid stability (%, red circle) were determined as described in the Methods section.

The batch production of LMG194 cells transformed with pDsbALCIo was performed at 37°C in the HDM medium, a balanced complex medium previously optimized for high cell density cultures in multiple microfermentors [16]. Addition of arabinose was performed at an OD_600 _value of 16, equivalent to 5.5 g dry cell weight (DCW) per liter, and the culture continued to grow during the next 8-h period (figure 4). The level of periplasmic LCI increased up to 5 h after induction, then decreased progressively. In contrast, the level of extracellular LCI increased continuously until a cultivation time of 8 h, resulting in most of the overproduced protein being found in the culture supernatants. This result suggested that a high concentration of periplasmic LCI is required before being released into the growth medium, presumably because of an increased permeability of the outer membrane. Although that the mechanism of this non- or semi-specific [2] protein secretion is still unclear, it was proposed that the high level production of secreted protein could inhibit the synthesis of outer membrane proteins, and compromise the permeability barrier of outer membrane. Therefore, we checked the viability of cells during cultivation in the fermentor, and in parallel experiments we monitored CFU counts for plasmid loss. The data shown in figure 5 indicate that about 90% of cells were still alive and able to form colonies on selective plates. The accumulated LCI in the growth medium during high cell density culture did not result from cell lysis or death. Finally, after 8 h of induction, about 470 mg of active LCI could be purified from 1 liter of culture by a single step reverse-phase chromatography.

## Conclusion

In this study, we found that *E. coli *codon optimization in the LCI gene when combined to the signal sequence of DsbA allowed the production/purification of 470 mg of active LCI per liter of culture. While codon usage may be an important criterion for translation rate [17,18] and/or protein folding [18-20], our studies indicate that, besides the nature of signal sequence, it is also an important parameter to ensure an efficient export of heterelogous precursors. If the nature of signal sequence determines the targeting pathways [21], the correct combination of both parameters appears to be necessary for optimal coupling of translation to protein translocation in *E. coli*.

## Methods

### Bacterial strain and plasmids

The *E. coli *LMG194 strain [F^- ^Δ*lacX74 galE galK thi rpsL *Δ*phoA *Δ*ara714 leu*::Tn*10*] carrying the *araBAD *deletion [12] was used as the expression host throughout the experiments. Recombinant DNA manipulations were performed as described in established protocols [22]. Plasmid pLCB was constructed in two steps from the pBAD33 expression vector [12]. First, the residual *bla *sequence was deleted by *Bgl*I-*Tth*111I digestion and filling in with Klenow fragment. Second, a DNA fragment which contained the Shine-Dalgarno sequence comprising an ATG start codon within a *Nde*I site from the pIVEX2.3MCS vector [23] was amplified using 5'-AAGAGCTCGAATTCCATATGTATATCTCCTTGCTAGCCCAAAAAAACGGGTATGG-3' and 5'-GTAACAAAGCGGGACCAAAGCC-3' as primers, and pBAD33 as DNA template. The PCR product was digested with *Mlu*I and *Sac*I, and cloned into the same restriction sites of the previous pBAD33 derivative. The structure of the resulting plasmid was confirmed by sequencing and designated as pLCB. The mature LCI sequence was codon optimized for *E. coli *expression and chemically synthesized by Geneart (Regensburg, Germany). The substitution of *malE *or *dsbA *signal sequence was generated by overlap extension PCR as previously described [24].

### Growth conditions

For shake flask cultures, cells were grown in 100 ml of LB medium supplemented with chloramphenicol (30 μg.ml^-1^). Induction of the *araB *promoter was accomplished by addition of L-arabinose to a final concentration of 0.2%. After 6 h at 37°C, cells were harvested by centrifugation at 6,000 rpm for 15 min. For high cell density cultures, bacteria were grown in a Sartorius Biostat B^® ^2-L fermentor at 37°C. The aeration rate and stirrer speed were regulated to keep the dissolved oxygen concentration at 60% of its saturation value. Precultures (80 ml) were prepared in shake flasks at 37°C to mid-log phase, and then added into the fermentor containing 800 ml of the HDM medium [16] supplemented with chloramphenicol (30 μg.ml^-1^). Induction was accomplished by addition of L-arabinose (0.5%). Cell biomass was monitored by measuring both the optical density at 600 nm (OD_600_) and dry cell weight (DCW) as previously described [25]. Cell viability was determined by using the LIVE/DEAD BacLight kit (Invitrogen) in combination with flow cytometry as described by the manufacturer [26]. Plasmid stability was assessed by plating properly diluted amounts of culture samples on LB-agar plates containing 0.5% arabinose without antibiotic and with 30 μg.ml^-1 ^chloramphenicol. After overnight growth at 37°C the numbers of colony forming unit (CFU) were determined.

### Cell fractionation and protein assays

Cells carrying the pLCB derivatives, normalized to the same OD_600_, were fractionated by spheroplast preparation as previously described [27]. To analyse secreted LCI in the culture media, culture supernatants were applied to SepPak Plus C18 cartridges (Waters) pre-equilibrated by 10% acetonitrile. Then, the columns were washed with 10% acetonitrile, and proteins were eluted by 30% isopropanol. Total protein content was determined by the Bradford assay using bovine serum albumin as a standard. Cellular fractions were separated on 10% Bis-Tris polyacrylamide NuPage gels (Invitrogen), and proteins were visualized by Coomassie blue staining. For quantitative analysis, gels were scanned with Gel Doc XR imaging system (Biorad).

### LCI purification

After cultivation, cells were centrifuged as described above and supernatants were filtered through a 0.22 μm syringe filter (Millipore). LCI was purified by reverse phase chromatography using a Ultimate 300 HPLC system (Dionex) and a Vydak C4 column, with a linear gradient ranging from 20 to 80% acetonitrile at a flow rate of 1 ml.min^-1 ^as previously described [9]. To quantify the concentration of native LCI found in periplasmic and culture supernatants, a calibration curve was constructed by using purified active protein as a standard. The LCI activity was assayed using the Carboxypeptidase A assay kit (Sigma Aldrich) in 50 mM Tris-HCl buffer, pH 7.5; containing 100 mM NaCl.

## Competing interests

The authors declare that they have no competing interests.

## Authors' contributions

JMP and JMB designed and performed experiments, interpreted the data and wrote the manuscript.
